# Metal-Free Click Modification of Triple Bond-Containing Polyester with Azide-Functionalized Vegetable Oil: Plasticization and Tunable Solvent Adsorption

**DOI:** 10.1021/acsomega.2c01525

**Published:** 2022-06-28

**Authors:** Karen Cangul, Emrah Cakmakci, Ozgun Daglar, Ufuk Saim Gunay, Gurkan Hizal, Umit Tunca, Hakan Durmaz

**Affiliations:** † Department of Chemistry, Istanbul Technical University, Istanbul 34469, Turkey; ‡ Department of Chemistry, Marmara University, Istanbul 34722, Turkey

## Abstract

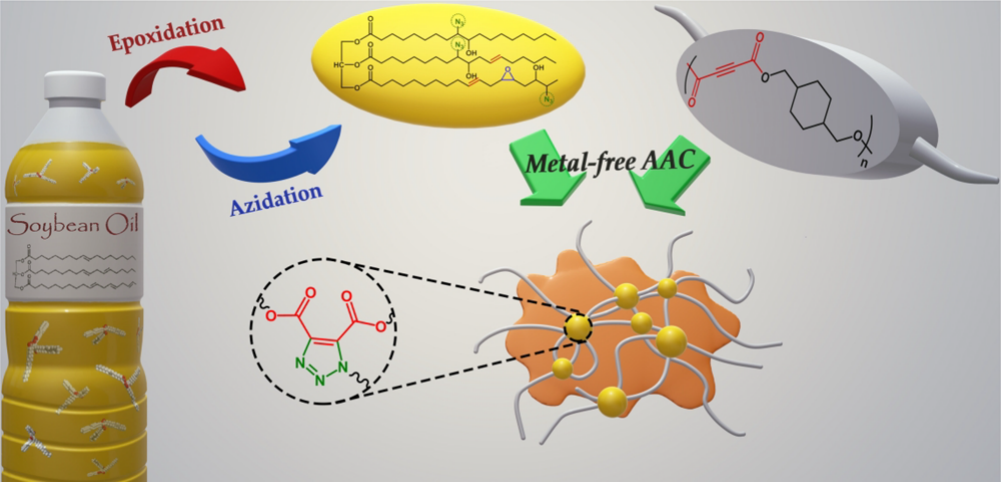

Pressure from environmental nongovernmental organizations and the public has accelerated research on the development of innovative and renewable polymers and additives. Recently, biobased “green” plasticizers that can be covalently attached to replace toxic and migratory phthalate-based plasticizers have gained a lot of attention from researchers. In this work, we prepared an azide-functionalized soybean oil derivative (AzSBO) and investigated whether it can be used as a plasticizer. We covalently attached AzSBO to an electron-deficient triple-bond-containing polyester via a metal-free azide–alkyne click reaction. The thermal, mechanical, and solvent absorption behaviors of different amounts of azidated oil-containing polyesters were determined. Moreover, the plasticization efficiency of AzSBO was compared with the commercial plasticizers bis(2-ethylhexyl) phthalate and epoxidized soybean oil. At relatively lower AzSBO ratios, the degree of cross-linking was higher and thus the plasticization was less pronounced but the solvent resistance was significantly improved. As the ratio of AzSBO was increased, the glass transition temperature of the pristine polymer decreased up to 31 °C from 57 °C. Furthermore, the incorporation of AzSBO also improved the thermal properties and 20% AzSBO addition led to a 60 °C increase in the maximum weight loss temperature.

## 1 Introduction

The ever-increasing environmental pollution problems associated with the use of petroleum-based resources for the manufacturing of plastic materials and the depletion of fossil raw materials gave rise to the concept of Green Chemistry at the end of the 1990s and have prompted researchers to seek safe and sustainable alternatives.^1−4^ Biobased renewable building blocks such as vegetable oils, starch, cardanol, lignin, rosin, furan, terpenes, and so on are promising candidates for the preparation of thermoplastic or thermoset polymers. Among them, vegetable oils that are the triglycerides of fatty acids with glycerol, are one of the most preferred and prominent feedstocks because of their high abundance, low cost, and inherent biodegradable nature and are suitable for modification by many different chemical routes.^5−10^ Although vegetable oils can be used for functionalization, derivatization, and polymer preparation without disturbing the triglyceride structure; glycerol, monoglyceride, diglyceride, or fatty acids can be used for the same goal. Indeed, plant oil-based alkyd resins that are prepared by using monoglycerides have been used as binders for paints and coatings for about 100 years.^11^

In recent years, there has been a growing trend in the development of vegetable oil-based precursors as alternative polymer materials.^12^ In most cases, plant oils are first subjected to various modifications.^13^ For instance, epoxidation of double bonds of vegetable oils has become a routine modification route.^14^ The epoxidized vegetable oils are cured thermally with amines,^15^ or directly via photoinitiated cationic photopolymerization to synthesize biobased thermoset materials^16^ or they are reacted with acrylic acid and used in photocurable coating formulations.^17^ Modern techniques such as thiol–ene addition reactions,^18,19^ azide–alkyne click protocols,^20^ and acyclic diene metathesis^21^ have also been applied to functionalize/polymerize vegetable oils or their constituents.

Aside from these direct uses of vegetable oils for the preparation of plastic materials, lately, derivatives of vegetable oil have been used as additives or agents for a wide range of applications including the modification of commercial polymers. Vegetable oils were used as plasticizers, lubricants, stabilizers, processing aids, surfactants, etc.^22−24^ Especially, much effort has been devoted to the use of vegetable oils as plasticizers, particularly for polyvinyl chloride (PVC).^25−29^ To prevent the migration of the plasticizers, vegetable oil-based compounds were also covalently attached to PVC via azide–alkyne click reactions.^30−32^

**Scheme 1:**
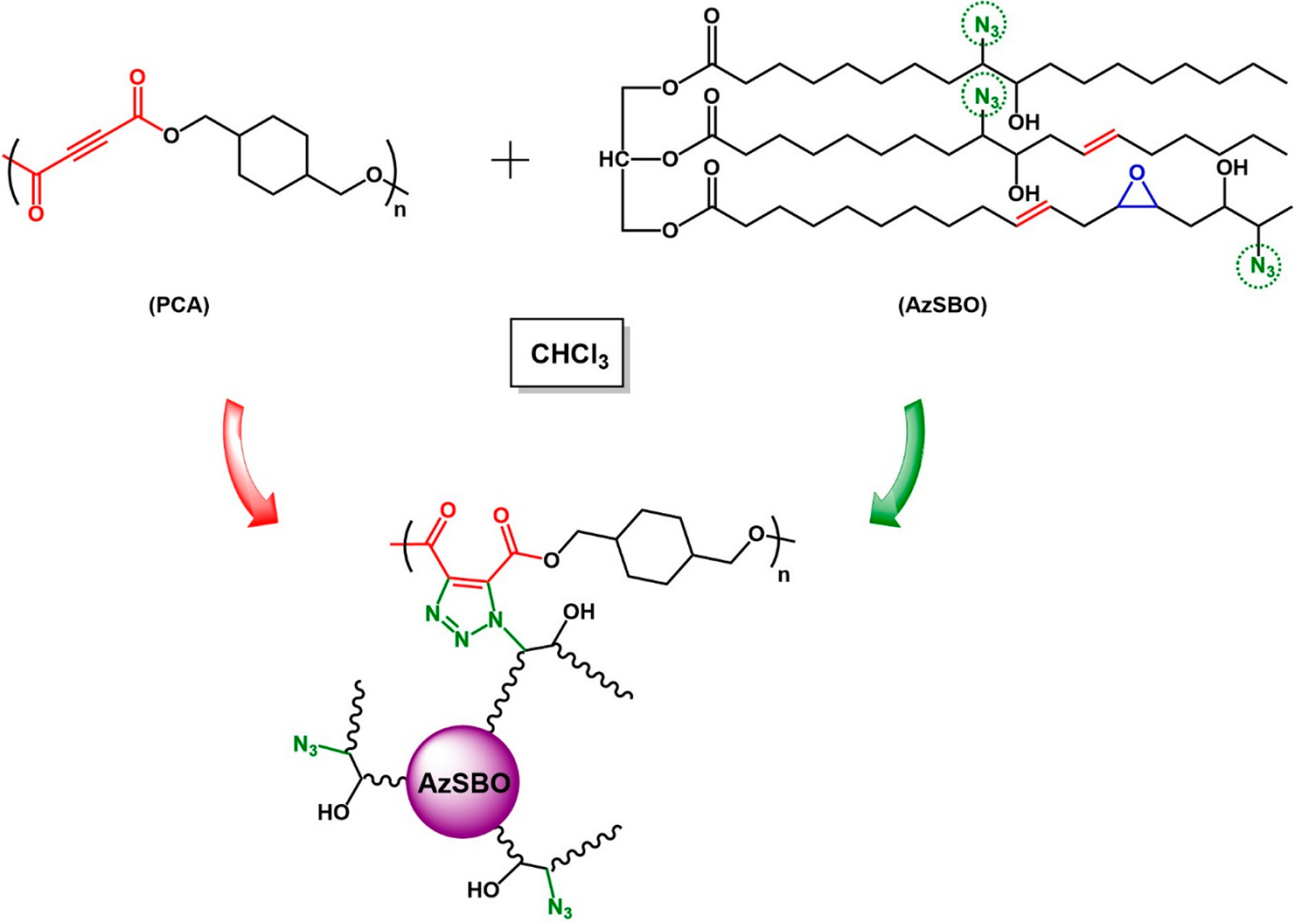
Schematic Representation of the Modification of PCA with AzSBO

Recently, our group has focused on the synthesis, modifications, and applications of electron-deficient triple bond-containing polyesters.^33−40^ The activated alkyne bonds on the polyester backbone made it possible to perform reactions such as aza-Michael, thiol-Michael, Diels–Alder, azide–alkyne click reactions readily under mild conditions, in the absence of metal catalysts, and high yields. Notably, the modification of the triple bond-containing polyesters via the metal-free azide–alkyne cycloaddition is an intriguing feature for the elimination of copper catalysts and benign reaction conditions. Herein, we report the modification of polyester bearing alkyne groups; namely, poly(1,4-cyclohexanedimethylene acetylene dicarboxylate) (PCA), with azide-functionalized soybean oil which we aimed to use as a renewable plasticizer (Scheme 1). It is worth mentioning here that PCA is a reactive triple bond-containing cycloaliphatic polyester with good film forming properties and a unique platform to be modified via various amines, thiols, azides, etc.^33,36,37,39^ PCA and similar electron-deficient triple bond-containing polyesters can be regarded as a modern alternative to conventional unsaturated polyesters. So far, we have utilized PCA for the preparation of polyhedral oligomeric silsesquioxanes-containing hybrid networks,^36^ hydrophobic electrospun surfaces,^37^ and silica nanoparticle-containing hybrid nanocomposites.^39^

## 2 Experimental Section

### 2.1 Materials

Raw soybean oil (SBO) was obtained from a local company. Formic acid, hydrogen peroxide (30 wt %), sodium chloride, ethyl acetate, anhydrous sodium sulfate, sodium bicarbonate, and chloroform were purchased from Sigma-Aldrich and used as received. PCA (*M*_n_ = 24.9 kDa, *M*_w_ = 70.3 kDa, and *Đ* = 2.82) was synthesized according to our previous publications.^33,36^ DOP (bis(2-ethylhexyl) phthalate) was obtained as a gift from Plastifay (Turkey).

### 2.2 Characterization

FTIR spectra were recorded on a Cary 630 FTIR (Agilent Technologies) instrument over the range 4000–600 cm^–1^. ^1^H (500 MHz) was recorded using an Agilent VNMRS 500 instrument in CDCl_3_. Gel permeation chromatography (GPC) measurements were carried out with an Agilent instrument (model 1100) with a pump, refractive index, and UV detectors and four Waters Styragel columns (HR 5E, HR 4E, HR 3, HR 2; 4.6 mm internal diameter, 300 mm length, packed with 5 μm particles). The effective molecular weight ranges of columns are 2000–4 000 000, 50–100 000, 500–30 000, and 500–20 000 g/mol, respectively. THF was used as eluent at a flow rate of 0.3 mL/min at 30^ο^C, and 2,6-di*tert*-butyl-4-methylphenol (BHT) was used as an internal standard. The number-average molecular weights (*M*_n_) and dispersities (*Đ*) of the polymers were calculated based on linear polystyrene (PS) standards (Polymer Laboratories). Elemental analyses were carried out using a LECO CHN 932. Differential scanning calorimetry (DSC) experiments were performed under a nitrogen atmosphere on the PerkinElmer Pyris Diamond DSC apparatus. Samples were kept at 30 °C for 2 min and then heated to 100 °C with a heating rate of 20 °C/min. After holding 5 min at this temperature, samples were cooled to −10 °C with a cooling rate of 20 °C/min, followed by maintaining at this temperature for 2 min. Finally, they were reheated to 100 °C with a heating rate of 20 °C/min. Data from the second heating cycle were reported. Thermogravimetric analyses (TGA) of the films were performed by using a PerkinElmer thermogravimetric analyzer (Pyris 1 TGA model). Samples were run from 30 to 600 °C with a heating rate of 10 °C/min under a nitrogen atmosphere. Dynamic mechanical analysis (DMA) was performed on a PerkinElmer DMA 8000 analyzer in the tension mode. Samples (40× 10 × 0.1 mm) were clamped, and strain was applied at a frequency of 1 Hz and a heating of 3 °C/min from −20 to 100 °C. The water contact angles (CA) of the polyesters were determined on a Kruss (Easy Drop DSA-2) tensiometer. Measurements were made using 3–5 μL drops of distilled water. For each sample, at least three measurements were made, and the average was taken. Tensile tests were performed at room temperature on a Materials Testing Machine Z010/TN2S, using a crosshead speed of 10 mm/min on rectangular specimens. An average of at least three measurements is reported. The epoxy equivalent weight (EEW, mol/100 g) was determined by the hydrochloric acid (HCl)-acetone method.^41,42^

### 2.3 Epoxidation of Soybean Oil (ESBO)

Soybean oil was epoxidized according to the literature.^43−45^ Briefly, 50 g of dry soybean oil was added to a two-neck round-bottom flask equipped with a pressure-equalizing dropping funnel and a condenser. Three grams of formic acid was added to the soybean oil and the mixture was heated to 45 °C. Under stirring, hydrogen peroxide (10 g) was then added drop by drop in 1 h. After the addition was completed, the temperature was raised to 65 °C and the reaction was allowed to proceed for 6 h at this temperature. Afterward, the reaction flask was cooled to room temperature. 100 g of ethyl acetate was added to the reaction mixture and then transferred to a separatory funnel. The solution was first washed several times with water, followed by washing with dilute sodium bicarbonate solution (5%). The organic phase was separated, dried with anhydrous magnesium sulfate, and filtered, and ethyl acetate was removed via a rotary evaporator.

### 2.4 Azidated Soybean Oil (AzSBO)

**Scheme 2:**
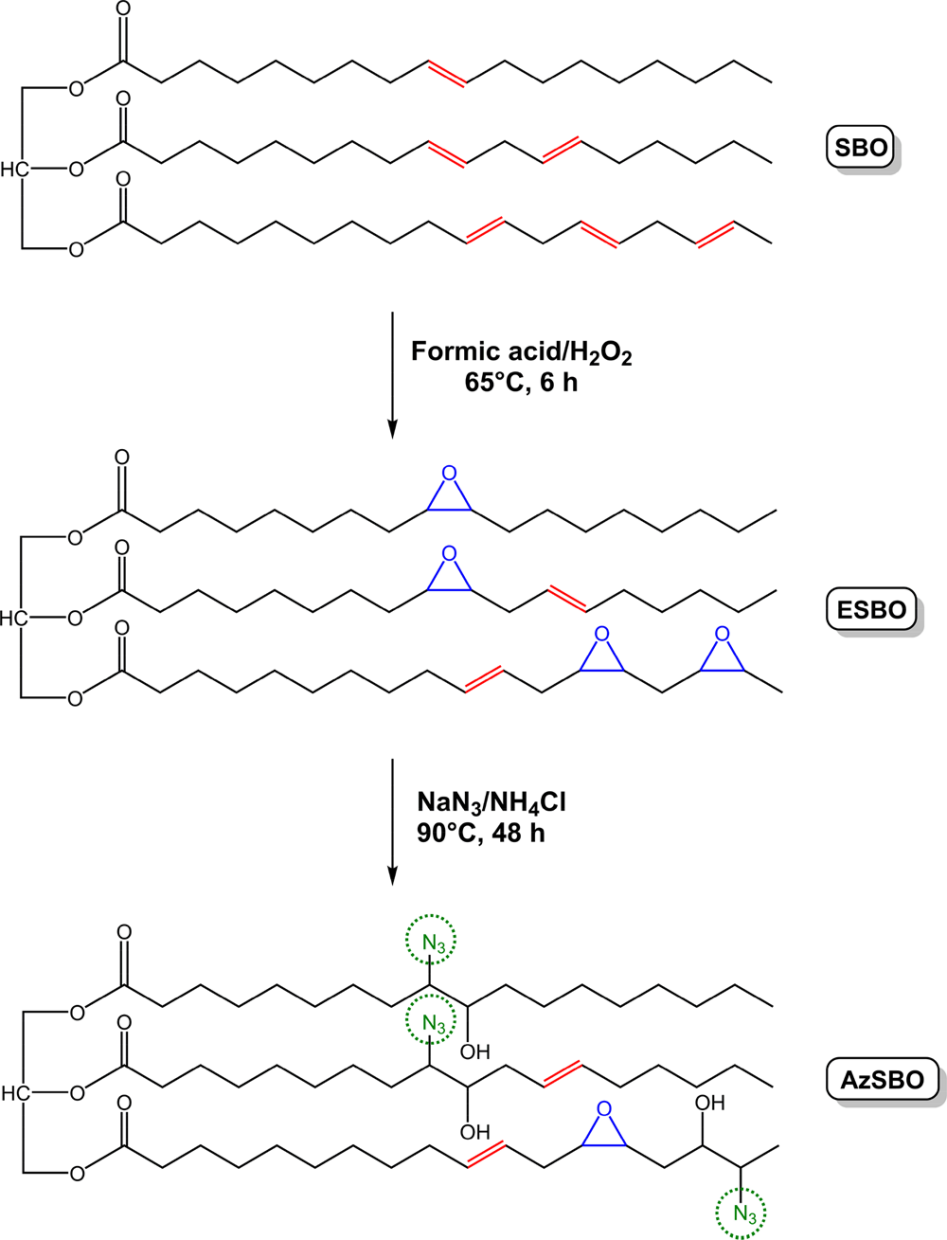
Synthetic Route for the Preparation of ESBO and AzSBO

Azide-functionalized soybean oil was prepared by the ring-opening of the epoxide groups of the soybean oil with sodium azide.^46,47^ Twenty grams of epoxidized soybean oil was mixed with 6 g of sodium azide, 4 g of ammonium chloride, water (20 mL), and ethanol (50 mL) in a round-bottom flask. A condenser was attached to the flask and the mixture was refluxed at 90 °C under constant stirring for 48 h. After cooling this mixture to room temperature, 100 mL of water was added and azidated soybean oil was extracted with dichloromethane (20 mL × 4). The organic phase was dried over anhydrous magnesium sulfate. After filtration, the organic phase was evaporated and AzSBO was obtained as a highly viscous brown liquid. The nitrogen content of the AzSBO was determined by elemental analysis. The synthesis of ESBO and AzSBO is illustrated in Scheme 2.

### 2.5 Metal-Free Modification of PCA by AzSBO

One gram of PCA was dissolved in 2 mL of CHCl_3_ and then the required amount of AzSBO (20, 40, and 60% with respect to the amount of PCA) was added. This mixture was stirred for 5 min and then the mixture was poured on Teflon molds and gradually heated to 60 °C to evaporate CHCl_3_ and catalyze the azide–alkyne click reaction. Neat PCA films were also prepared similarly without using the AzSBO. The films were named PCAX, where X symbolizes the percentage of the added AzSBO.

For comparison, ESBO- or DOP-containing PCA films were prepared similarly as described above. Two-tenths of a gram of ESBO or DOP was added per gram of PCA.

## 3 Results and Discussion

### 3.1 Characterization of AzSBO

**Figure 1:**
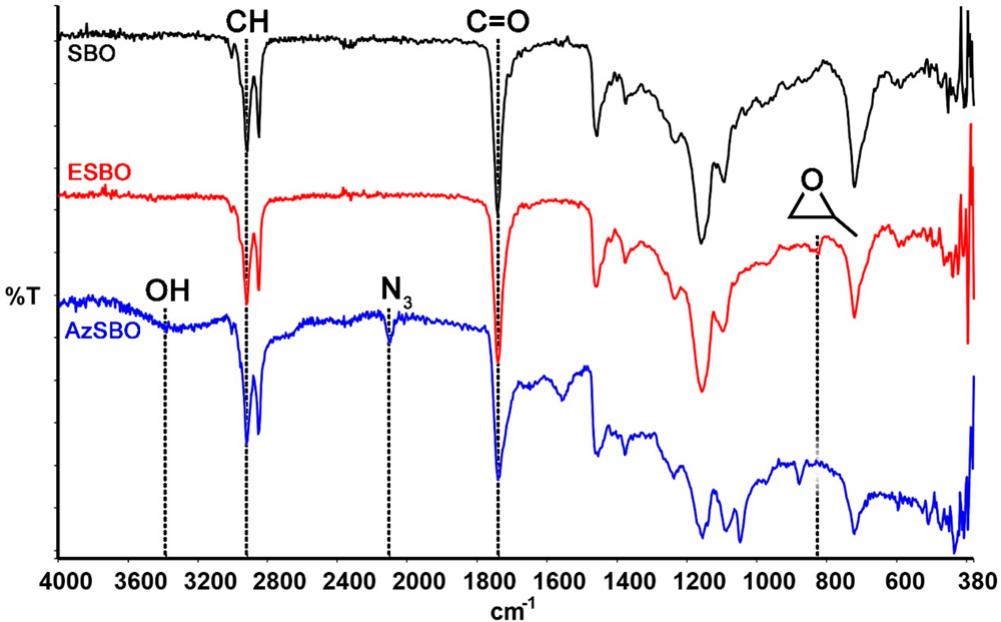
FTIR spectra of SBO, ESBO, and AzSBO.

The AzSBO was prepared from the reaction of NaN_3_ with ESBO. The AzSBO was characterized structurally with FTIR and ^1^H NMR spectroscopy. Figure 1 displays the FTIR spectra of pristine soybean oil, ESBO, and AzSBO, respectively. The FTIR spectrum of SBO exhibited the characteristic bands at 1740, 2850, 2920, and 3010 cm^–1^ which were ascribed to the carbonyl groups, symmetric and asymmetric stretching of −CH– bonds, and to the carbon–hydrogen stretching vibrations of the alkene double bonds within the triglyceride structure, respectively. After the epoxidation reaction, new bands appeared at 847 and 822 cm^–1^, which correspond to the epoxy groups, whereas the intensity of the alkene double bonds at 3010 cm^–1^ slightly declined.^42,43,46^ Finally, the ring-opening of the epoxide groups with NaN_3_ led to a substantial disappearance of the characteristic epoxide bands at 847 and 822 cm^–1^ and the formation of new hydroxyl bands at around 3500 cm^–1^ and azide bands at 2100 cm^–1^, as can be seen from the FTIR spectrum of AzSBO. All these FTIR findings are in good accordance with the literature and confirm the structure of AzSBO.^46,48−50^

**Figure 2:**
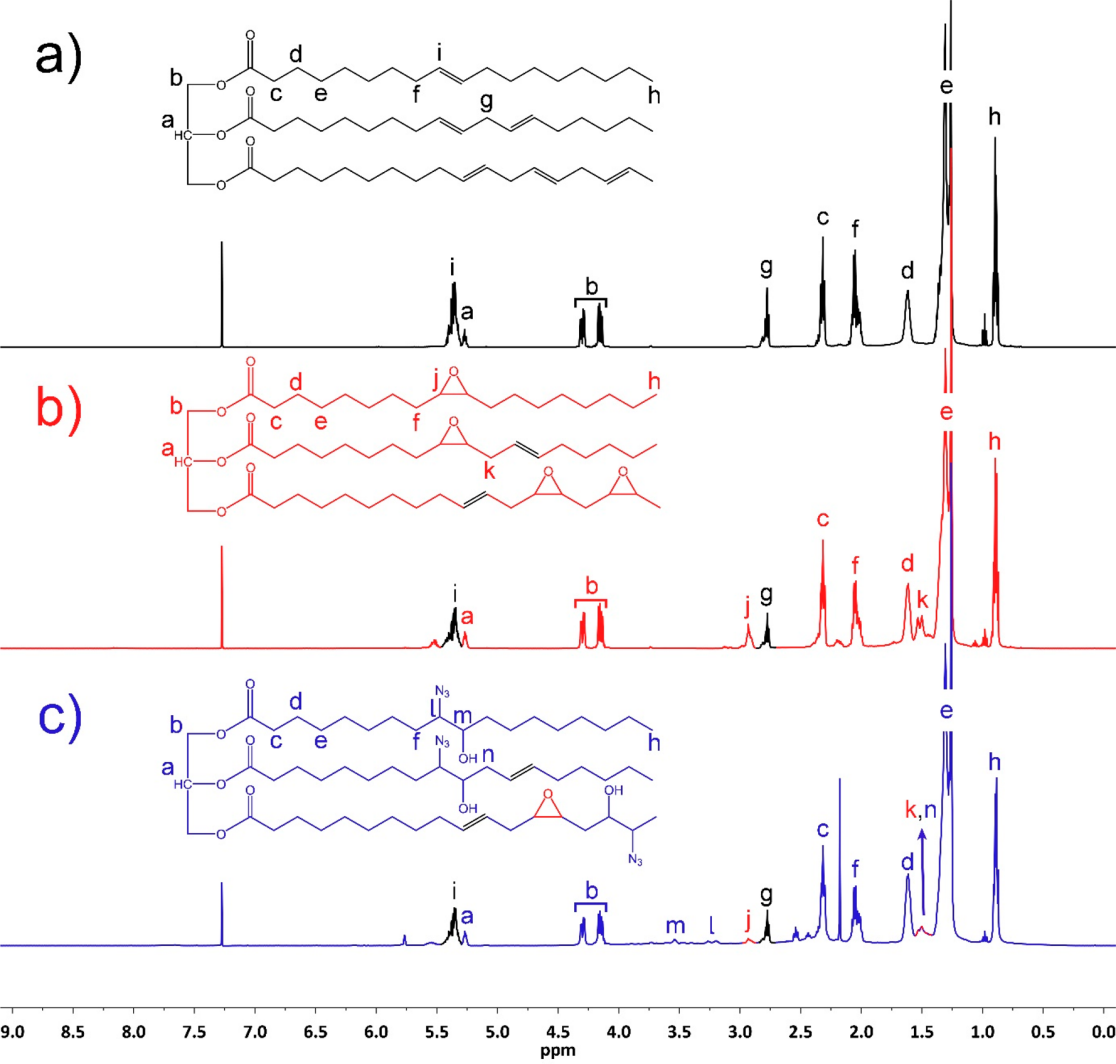
^1^H NMR spectra of (a) SBO, (b) ESBO, and (c) AzSBO.

The structures of SBO, ESBO, and AzSBO were also investigated by ^1^H NMR spectroscopy (Figure 2). The characteristic methine (a) and methylene (b) protons of SBO’s glycerol unit were observed at 5.2 and 4.3–4.1 ppm, respectively. These peaks did not change after epoxidation and azidation reactions. The peak at around 5.4 ppm was attributed to the internal alkene double-bond protons of SBO. Upon epoxidation, the intensity of this peak was decreased, indicating that the reaction took place. Furthermore, a new peak appeared at 3.0–2.8 ppm that was attributed to the characteristic protons of the epoxide rings.^45,47,48^ After the azidation reaction, these epoxy proton peaks almost disappeared because of the ring-opening reaction and shifted to 3.2 and 3.5 ppm.^47,51^ The former peak stemmed from the protons adjacent to the azide groups and the latter peak was due to the protons neighboring the −OH groups. All these findings prove that the AzSBO was synthesized successfully.

Here, we must note that we deliberately aimed for low epoxide conversion and thus a lower azide group content to minimize the probability of cross-linking. We calculated the epoxidation value by titration and found it as 0.2529 (EEW = 395). On the basis of ^1^H NMR^52^ we calculated the degree of epoxidation as 25.75%. A fully epoxidized SBO was reported to have an EEW of 231.^53^ Therefore, it can be said that the extent of epoxidation was low, as expected. Moreover, the nitrogen content of the AzSBO was found to be as low as 3.3% according to elemental analysis.

As noted previously, several alkyne-bearing plasticizers were synthesized and used for the plastification of PVC. Among the studies where vegetable oils were used as the building blocks for the synthesis of alkyne groups-containing clickable plasticizers, harsh, multistep reactions were conducted in the presence of copper catalysts.^30−32^ It must be noted that in some of these works phosphorylated plasticizers were synthesized, but even when the phosphorylation step is ignored, at least three or more steps are involved. Thus, the method suggested in this work is relatively simple compared to several strategies proposed in the literature. In addition, in most of the previous attempts, rather than the vegetable oil itself, vegetable oil-derived fatty acids or fatty acid esters were used, generating glycerol as waste. Here, in some sense, the applied strategy also valorizes glycerol by preserving the vegetable oil’s triglyceride structure.

### 3.2 Physical Appearance and the Structural Characterization of the AzSBO-Modified PCA

In this study, we mixed AzSBO with PCA at different weight ratios. Our preliminary trials with percentages above 60% of AzSBO resulted in mushy, tacky, and pastelike materials. Above this threshold value, the obtained materials displayed macroscopic phase separation. Furthermore, we were only able to prepare relatively thin films (>1 mm). A mushy appearance was also observed when we tried to prepare thicker samples. The synthesized AzSBO contains relatively fewer azide units and is a large molecule with sterically hindered azide functionalities; thus, rather than cross-linking of the PCA chains, modification from different points of the polymers is anticipated. Nevertheless, because AzSBO is multifunctional (contains many azide groups per triglyceride) the cross-linking of the PCA chains is unavoidable. One gram of PCA requires (Mru = 222 g/mol) 4.5 mmol of azide groups (0.189 g of nitrogen) for full cross-linking. Because the N% of AzSBO was found to be 3.3%, even at the highest AzSBO-containing formulation (0.6 g/1 g of PCA), the nitrogen content (0.0198 g) is far below the amount of nitrogen needed to consume all the triple bonds. This result is in line with our goal to use AzSBO as a plasticizer rather than a cross-linking agent.

**Figure 3:**
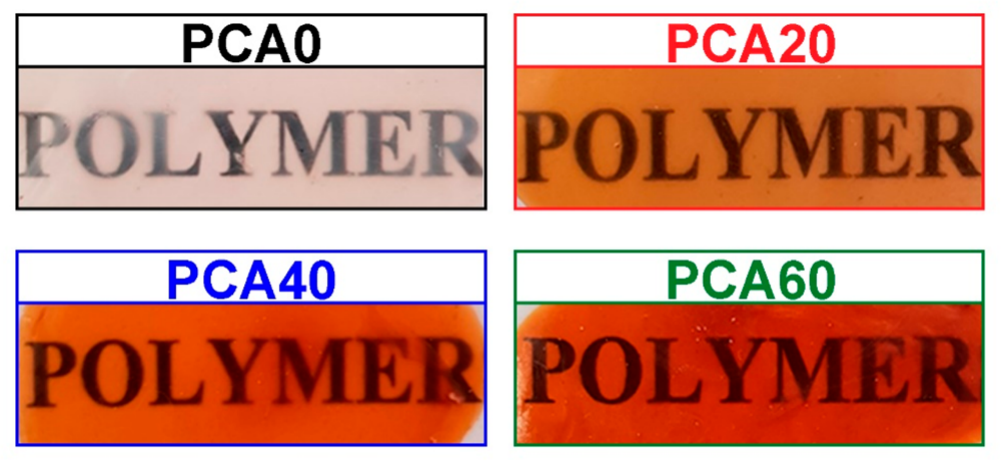
Digital photographs of PCA and AzSBO-modified films.

The photographs of the prepared films are supplied in Figure 3. it can be seen from this figure, pristine PCA produces colorless and transparent films. On the other hand, AzSBO-modified films were brown, reflecting the color of the AzSBO.

**Figure 4:**
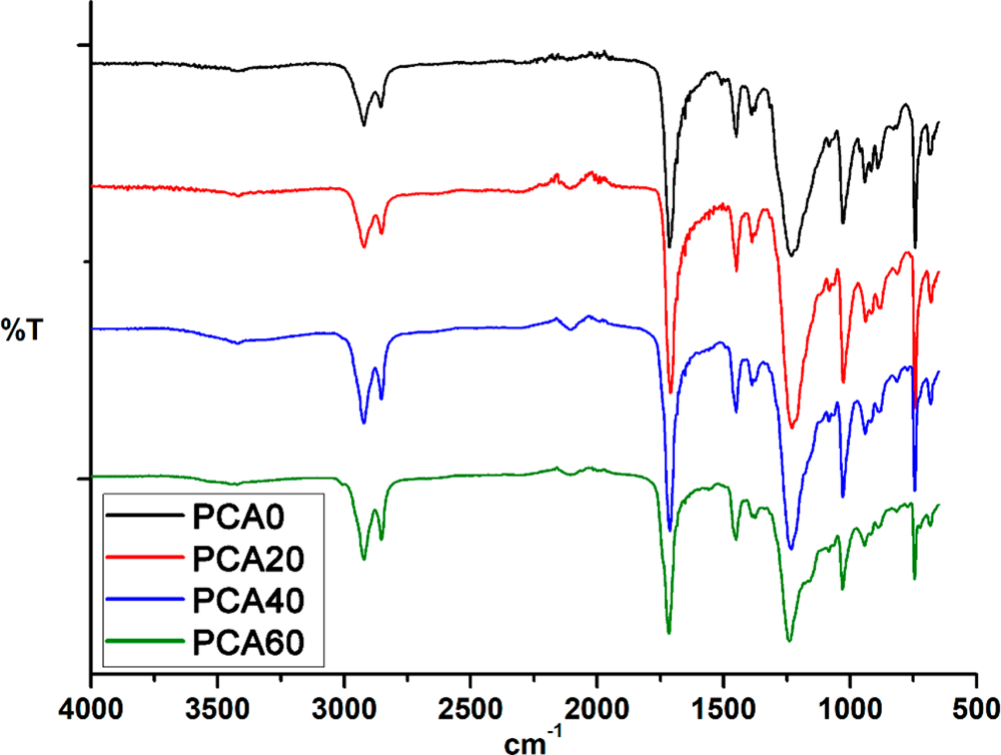
FTIR spectra of PCA and AzSBO-modified films.

The FTIR spectra of the AzSBO-modified PCA films are given in Figure 4. It can be seen from this figure that all films have similar spectra. The characteristic ester carbonyl band of PCA was observed at 1735 cm^–1^ and this band did not undergo any change upon the addition of AzSBO. The azide stretching vibrations can be seen as weak bands at around 2100 cm^–1^ in these spectra, indicating that not all azide groups were reacted but confirming the reaction of the azide groups of AzSBO with the triple bonds of PCA.

### 3.3 Wettability of the Polyester Films

**Figure 5:**
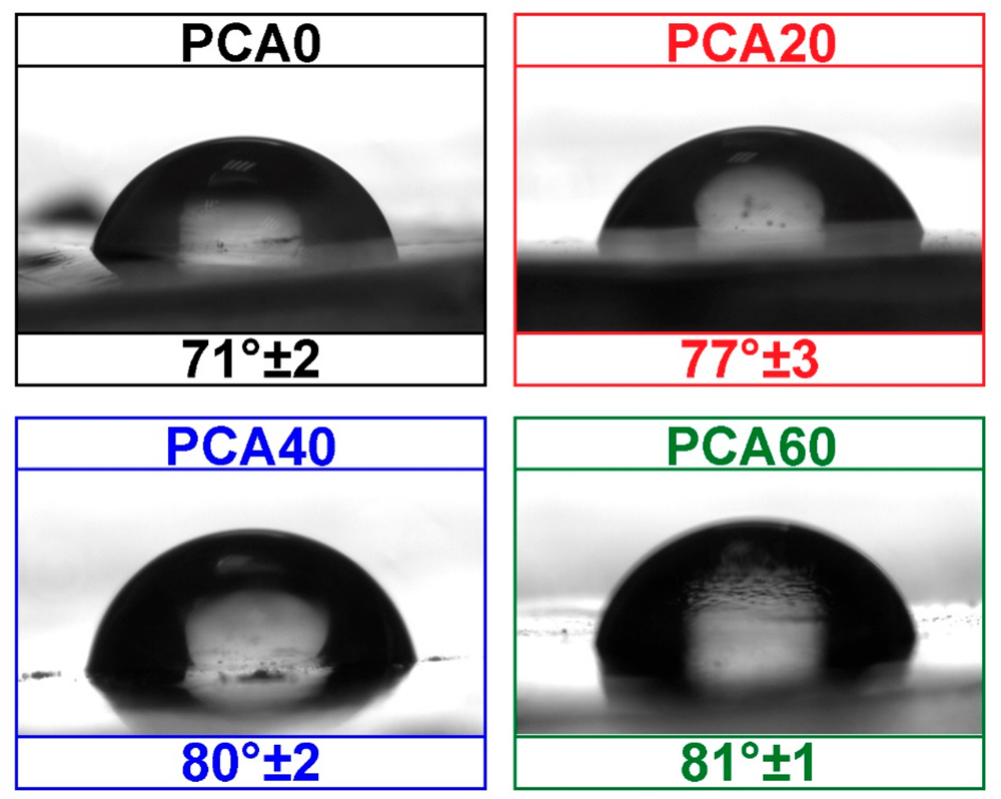
WCA values of the films.

The effect of the azidated vegetable oil on the surface wettability of the polyesters was investigated by measuring their WCAs (Figure 5). The WCA of PCA was found to be 71°, which is close to the WCA of PET. The incorporation of the AzSBO led to an increase in the WCA values of the modified films, which can be ascribed to the water-repellent nature of plant oils. For instance, 40% AzSBO addition increased the WCA to 81° ± 2. Thus, as expected, the AzSBO rendered PCA films hydrophobic. It must be noted here that when the AzSBO ratio was increased to 60%, it was observed that the WCA value did not change much. This can be attributed to the increased number of polar −OH groups and unreacted polar azide groups, which lead to a decline in the water repellency of the plant-oil-modified PCA.

### 3.4 Solvent Absorption Percentages of the Modified Polyesters

**Figure 6:**
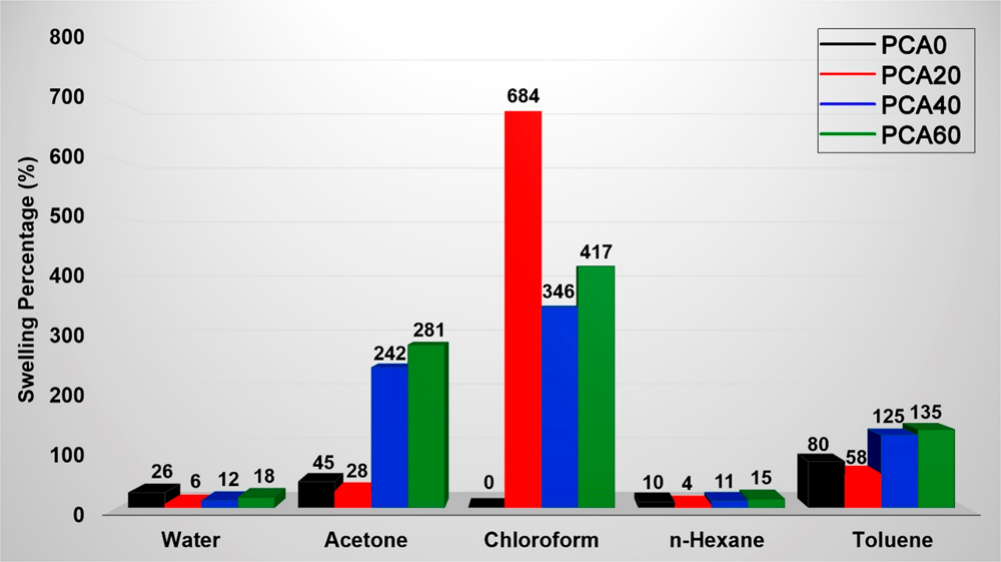
Swelling percentages of the polyester films in different solvents after 24 h. The swelling percentages in chloroform are reported for 3 h. The films were disintegrated in CHCl_3_ and slowly dissolved.

We measured the swelling behavior of the polyester films in different solvents (Figure 6). The films were cut into small pieces, dried in a vacuum oven at 40 °C for 24 h, and weighed. Dried pieces were then immersed into containers containing 10 mL of different solvents and kept for 24 h. At different time intervals, the appearance of the films was visually controlled and the swollen films were weighed.

Pristine PCA displayed a significant amount of water absorption (26%). When PCA was modified with 20% AzSBO, a dramatic decrease was observed for its water absorption value. Further AzSBO addition also improved the water absorptivity but the enhancement was found to be lower when compared to PCA20 and also as the amount of AzSBO increased to 60% from 40%, the solvent absorption percentage was increased (still lower than neat PCA). The initial decrease in water absorption indicates that at this AzSBO ratio films are relatively cross-linked, repelling the diffusion of water. On the other hand, the latter increase in water absorption can be explained by the decreased cross-linking density, leading to the exposure of the polar azide and hydroxyl groups which in turn contribute to the water absorption. For the organic solvents, a similar situation was encountered. For acetone, hexane, and toluene, solvents in which neat PCA is insoluble, first, the solvent absorption was decreased because of the relatively higher cross-linking density, and then the solvent absorption percentages were increased with an increasing amount of AzSBO because of the lightly cross-linked nature of PCA40 and PCA60 and due to the strong affinity of the vegetable oil-based AzSBO toward these solvents. Among these three solvents, films exhibited less affinity toward hexane and higher swelling in relatively polar acetone.

In the case of chloroform in which PCA was soluble, the films exhibited a rather peculiar behavior. The pristine PCA films (PCA0) were completely dissolved in CHCl_3_ within 15 min. Contrary to PCA0, AzSBO-modified films gained resistance to dissolution because of cross-linking and swelled in CHCl_3_ for at least 3 h. At the end of this period, swollen films started to break apart into small pieces and within 24 h completely dissolved. This finding is important since it reveals that the films were lightly cross-linked. As opposed to the swelling behaviors in other solvents, the swelling percentage of PCA20 was found to be higher than that of PCA40 and PCA60 despite its relatively higher cross-linking density. It is thought that this result stems from the ease of dissolution of PCA40 and PCA60 in chloroform. The relatively faster dissolution of PCA40 and PCA60 resulted in lower swelling percentages in CHCl_3_ when compared to PCA20, which was much more resistant and produced higher swelling ratios before breaking apart.

Finally, after 24 h, we must note that the swollen films in acetone were dried at 40 °C under vacuum and reweighed to determine whether the unreacted AzSBO passes to the solvent. The acetone soluble fractions of PCA20, PCA40 and PCA60 were found as 6.5%, 9.8% and 13.5%, respectively. Even though a biobased plasticizer is used in this work, the nonmigratory effect ensures long-term stability for the polymer. The bulky, sterically hindered, and low amount of azide groups-bearing AzSBO resulted in relatively fewer attachment points to the triple bonds of PCA which in turn led to some unreacted AzSBO.

### 3.5 Thermal Properties

**Figure 7:**
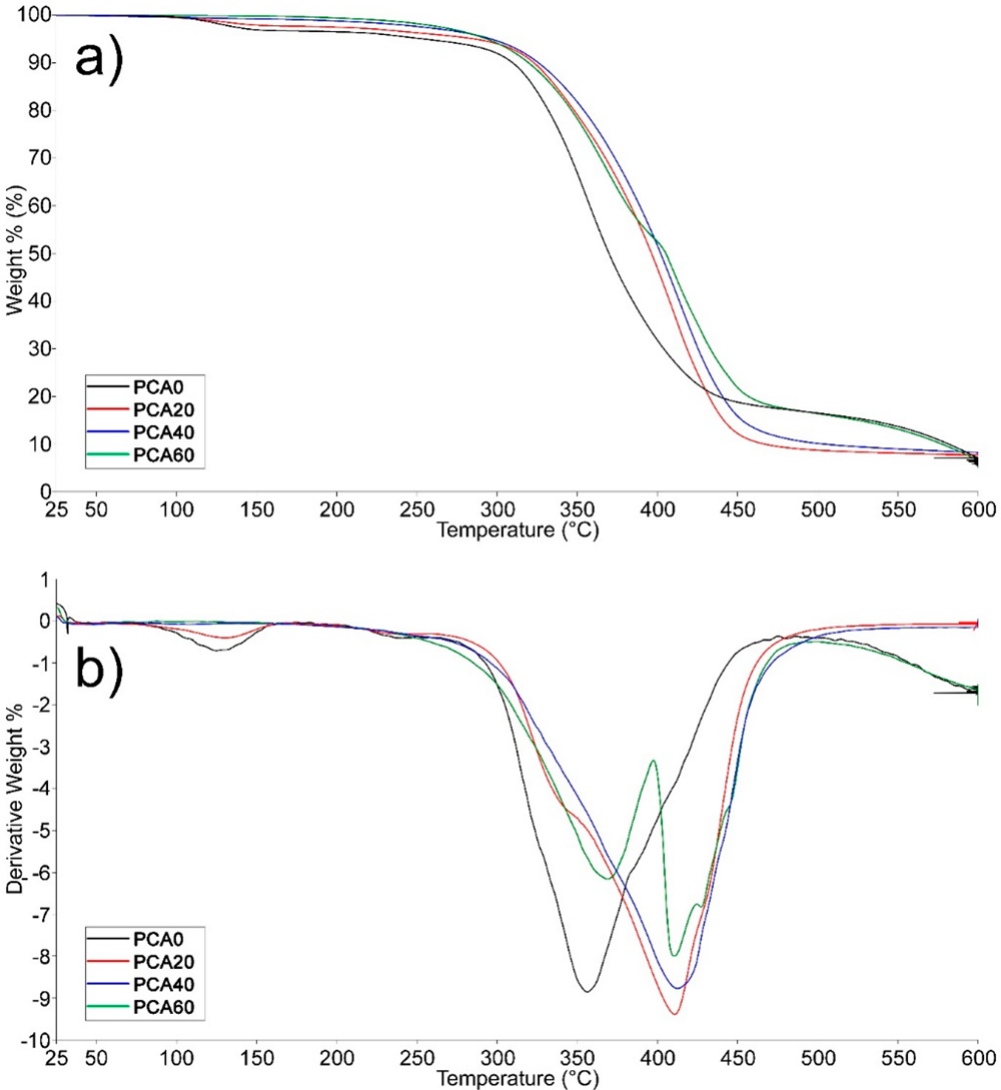
(a) TGA thermograms and (b) the derivative weight curves of PCA and the modified polyesters

The thermal stability of the PCA0 and the modified polyesters were determined by TGA. The TGA thermograms are presented in Figure 7 along with the corresponding derivative weight curves and the results are listed in Table 1.

**Table 1:** Thermal and Mechanical Properties of the Modified Polyesters

	*T*_max_^a^ (°C)	char (%)	*T*_g_^b^ (°C)	Young’s modulus (MPa)	tensile strength (MPa)	elongation at break (%)	*E*′_(20 °C)_^c^ (MPa)	ρx (× 10^3^ mol/cm^–3^)^c^
PCA0	350	5.56	57	531 ± 50	17.5 ± 2.4	8.8 ± 5.1	1.33	-
PCA20	410	7.7	48	153.2 ± 48	2.25 ± 0.25	12.6 ± 4.3	ND	ND
PCA40	410	8.1	40	113.5 ± 12	3.28 ± 0.16	76.4 ± 23	0.48	1.7
PCA60	350–410	5.139	31	75.58 ± 10	3.55 ± 0.59	22.46 ± 5.3	0.14	0.65

a *T*_max_ is the maximum weight loss temperature, which was determined from the maximum of the corresponding derivative curves.

b Determined by DSC.

c Determined by DMA.

PCA0 displayed a single-step degradation profile. The small amount of weight loss that occurred under 150 °C was attributed to the absorbed moisture, reflecting the hygroscopic nature of PCA. The main degradation temperature (*T*_max_) for PCA was found as 350 °C and the char yield at 600 °C was determined to be 5.56%. After modification and cross-linking, the thermal properties were improved. *T*_max_ values shifted to 410 °C and the char yields increased to 7.7 and 8.1% for PCA20 and PCA40, respectively. The improved thermal stability can also be ascribed to the formation of thermally stable, rigid triazole rings. PCA60, which produced the highest amount of acetone-soluble fraction, displayed a two-stage degradation profile and exhibited a relatively lower char yield. This result supports our view that the cross-linking density decreased with an increasing amount of AzSBO. The decreased cross-linking density adversely affected the thermal properties.

**Figure 8:**
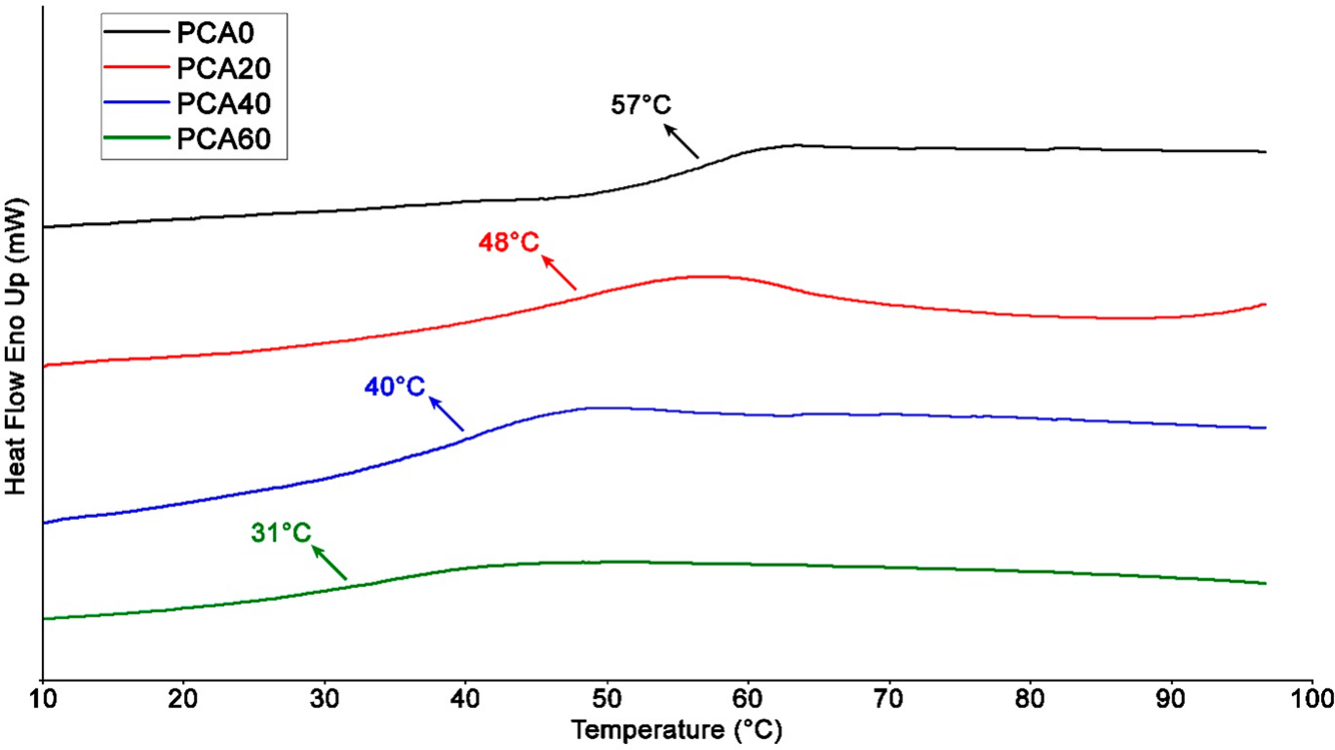
DSC curves of the polyesters.

The *T*_g_ values of the polyesters were determined by DSC and the DSC curves of the polyesters are presented in Figure 8. The *T*_g_ values were determined as 57, 48, 40, and 31 for PCA0, PCA20, PCA40, and PCA60, respectively (Table 1). These results clearly display the plasticization of PCA chains with the aid of AzSBO despite slight cross-linking. The covalently attached AzSBO molecules act as internal plasticizers while the unreacted AzSBO behaves like a common external plasticizer. AzSBO increases the distance between individual polymer chains and as a result, the free volume, mobility, and flexibility of the polyester chains increase. Najafi et al. prepared oleic acid-based internal plasticizers that were covalently attached to PVC chains.^54^ The *T*_g_ of the neat PVC decreased to 42.8 °C from 84 °C. In another work, Jia et al. prepared an amine-functional plasticizer from waste vegetable cooking oil, attached it to PVC via displacement of chlorines, and similarly, the *T*_g_ value was almost halved compared to PVC without plasticizer.^55^

We also prepared ESBO or DOP-containing PCA films and compared their plasticization performance with AzSBO. The DSC spectra of PCA/DOP and PCA/ESBO films are presented in Figures S1 and S2, respectively. As it can be seen from these spectra, both ESBO and DOP-containing PCA films displayed two endotherms; one approximately at the *T*_g_ value of the neat PCA (57 °C) and another *T*_g_ at a lower temperature. The lower *T*_g_ value for the PCA/DOP films was measured as 38 °C, whereas that for the PCA/ESBO films was found to be 36.5 °C. The presence of two *T*_g_ values indicate that both DOP and ESBO are not compatible with PCA. Although PCA20 encoded films that contain AzSBO as the plasticizer, displayed a relatively higher *T*_g_ value (48 °C) with respect to DOP and ESBO, they were found to be much more compatible with PCA. The relatively lower plasticization performance of AzSBO can be attributed to the introduced cross-linking sites. Fu et al. investigated the effect of dioctyl terephthalate (DOTP) and ESBO on PVC and found that they had similar plasticization efficiencies and when the same amount was added, the *T*_g_ for PVC/DOTP was determined to be 35.2 °C, whereas that for PVC/ESBO was 39.8 °C.^28^ Furthermore, when they used epoxidized castor oil (ECO) as plasticizer, the films displayed two *T*_g_ values, reflecting the incompatibility between ECO and PVC.

To gain more insight into the cross-linking and plasticizing effect, we investigated the thermomechanical properties of the AzSBO-containing PCA films. The storage modulus versus temperature plots of the polyester films are given in Figure S3 and the results are displayed in Table 1. Since we could not be able to prepare thick films, the DMA tan delta and loss modulus plots were too noisy and therefore they are not reported here. Furthermore, despite our several trials, we could not be able to record full plots for PCA20 encoded films, all samples broke during the tests. The cross-linking densities (ρx) of the polyesters were calculated by using the following equation according to the literature:^56^
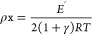
1where *E*′ is the rubbery storage modulus at *T*_g_ + 40 °C in MPa, *R* is the gas constant (8.3145 J/mol K), *T* is the temperature in K, and γ is Poisson’s ratio. γ was assumed to be 0.5.^56^ The *T*_g_ values were borrowed from the DSC results.

The DMA results apparently display the plasticizing effect of AzSBO. As the amount of the added AzSBO was increased, the storage modulus as well as the cross-linking densities of the polyester films decreased. Thus, it can be concluded that there is a good correlation between the observed decreasing trend in *T*_g_ values and the decreasing cross-linking densities. These results support our previous findings. As the cross-linking density of the polyester films increased, the restricted mobility led to relatively higher *T*_g_ values. For instance, the *T*_g_ of PCA40 was found to be 9° higher than that of PCA60 because of the more than 3-fold increase in the cross-linking density with respect to PCA60.

As it is known cross-linking and plasticization are two antagonistic effects. The greater the cross-linking density, the less the plasticization. Both the DSC and DMA results in this work clearly reflect this principle.

### 3.6 Mechanical Properties

**Figure 9:**
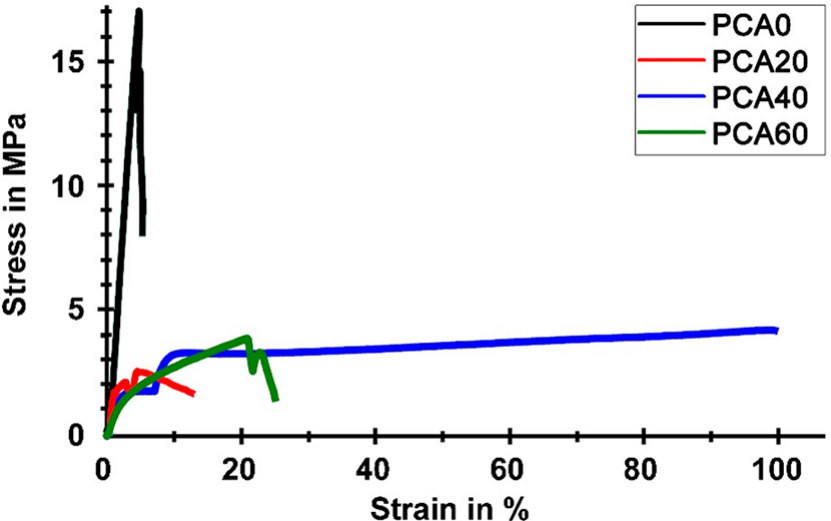
Representative stress–strain curves of the polyester films.

The tensile modulus, tensile strength, and elongation at break values of the PCA-based films were determined. The representative stress–strain curves are presented in Figure 9 and the results are collected in Table 1. Note that the data in Table 1 are the average of at least three different measurements. The Young’s modulus and the elongation at break value of PCA0 were found to be 531 MPa and 8.8%, respectively. The Young’s modulus of the PCA20 decreased dramatically to 153.2 MPa. The modulus decreased with a further increase in the amount of AzSBO. This result clearly shows that the films were lightly cross-linked and internally plasticized by AzSBO. It would be suitable to expect that the modulus would be higher or similar to PCA if the cross-linking density was much higher. Thus, based on these results we can conclude that in our case, modification of the PCA chains was prominent rather than cross-linking. Increased elongation at break values accompanied the decreased modulus values as a result of plasticization. The film specimens of PCA60 broke before they could get too long because of their low modulus values. The tensile strength values also declined when the modified vegetable oil was incorporated; however, the tensile strength values increased as the amount of AzSBO was increased.

## 4 Conclusions

As a continuation of our studies on triple-bond-containing polyesters, here we modified them with azidated vegetable oil and investigated the properties of the resulting polymers. AzSBO was attached to PCA chains via a metal-free azide–alkyne click reaction. PCA was modified with up to 60% AzSBO. After this threshold value, self-standing and visually uniform films cannot be produced. It turned out that the solvent absorption and the plasticization of the polyester chains could be controlled with the addition of different amounts of AzSBO. DSC and DMA results revealed that at lower ratios, AzSBO lead to cross-linking and thus the obtained films were resistant to solvent absorption and the plasticization was less pronounced compared to a relatively higher amount of AzSBO-containing films. As the amount of AzSBO was increased, the *T*_g_ values declined and the films became much more flexible because of the increased free volume and chain mobility. The addition of AzSBO also led to improvement in the thermal degradation temperatures due to cross-linking.

Here, the applied method is fast, straightforward, and effective compared to other methods in the literature on vegetable oil-based plasticizers. In addition, providing solvent resistance is another privilege. Yet, not all AzSBO reacted with PCA, thus the nonmigratory behavior was not revealed. Therefore, for future studies, we aim to develop new plasticizers by directing our work in a way that will further reduce migration.

## Supporting Information Available

DSC and DMA plots of the films (PDF)
