# Supplementary material for: Metal-Free Click Modification of Triple Bond-Containing Polyester with Azide-Functionalized Vegetable Oil: Plasticization and Tunable Solvent Adsorption
Source: ACS Omega. 2022 Jun 28;7(27):23332–41. doi: 10.1021/acsomega.2c01525 (PMC9281323; doi:10.1021/acsomega.2c01525)
Supplement: Supplementary file 1 — ao2c01525_si_001.pdf [file ao2c01525_si_001.pdf]

SUPPORTING INFORMATION

Metal-free click modification of triple bond-containing polyester with azide-functionalized vegetable oil: Plasticization and tunable solvent adsorption

Karen Cangul[1], Emrah Cakmakci[2*], Ozgun Daglar[1], Ufuk Saim Gunay[1*], Gurkan Hizal[1], Umit Tunca[1], Hakan Durmaz[1*]

[1]*Department of Chemistry, Istanbul Technical University, 34469 Istanbul/Turkey*

[2]*Department of Chemistry, Marmara University, 34722 Istanbul/Turkey*

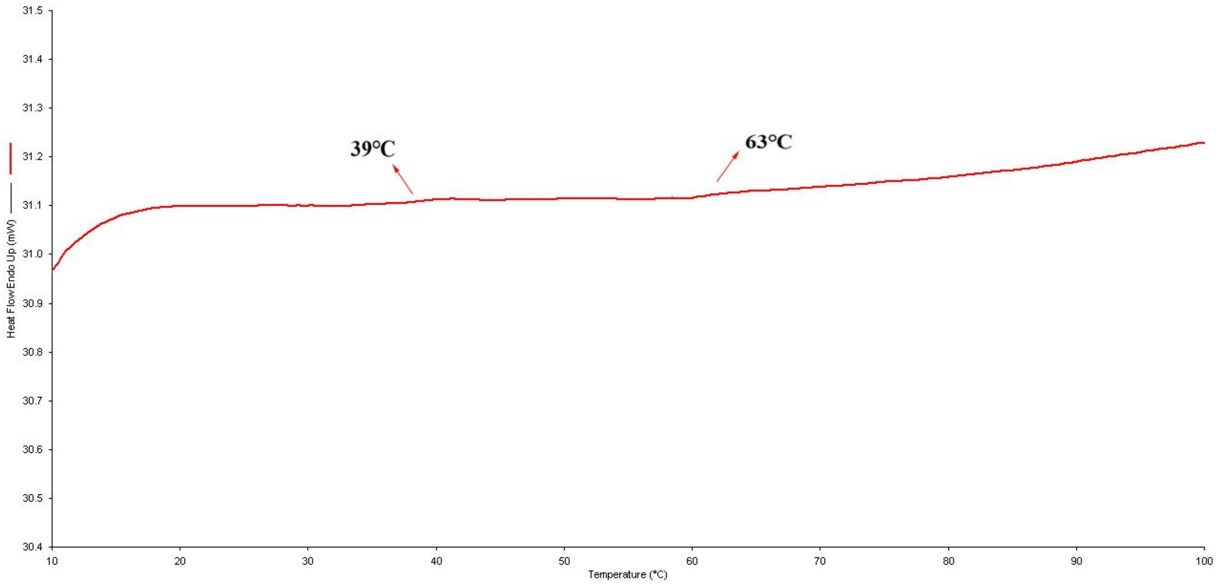

Figure S1. DSC spectrum of PCA/DOP films. The films were prepared by adding 0.2 grams of DOP to 1 gram of PCA.

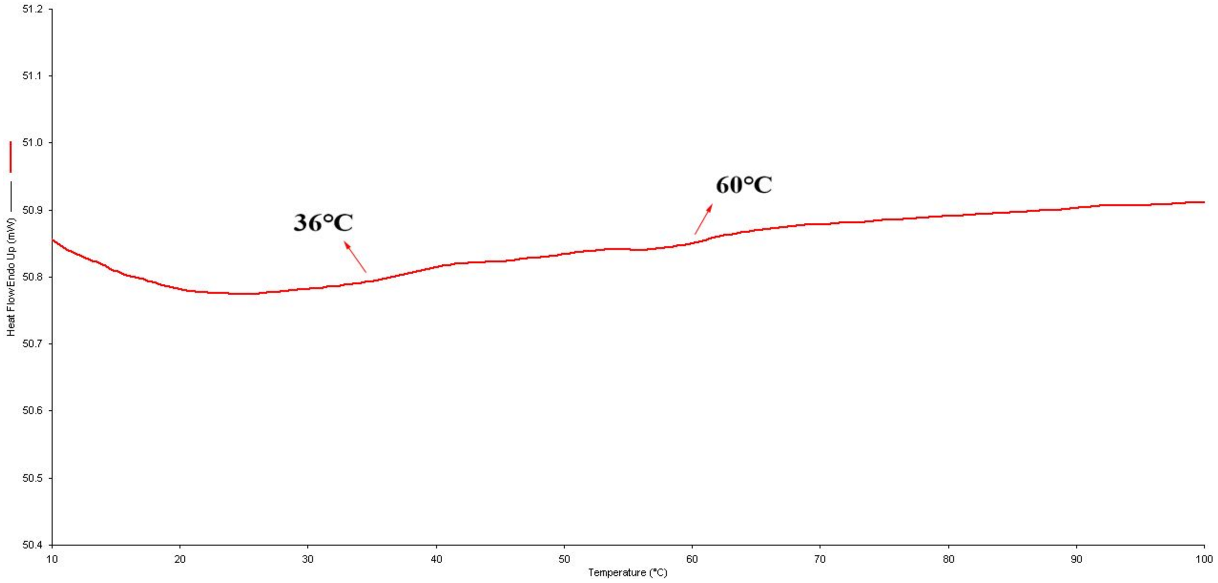

Figure S2. DSC spectrum of PCA/ESBO films. The films were prepared by adding 0.2 grams of ESBO to 1 gram of PCA.

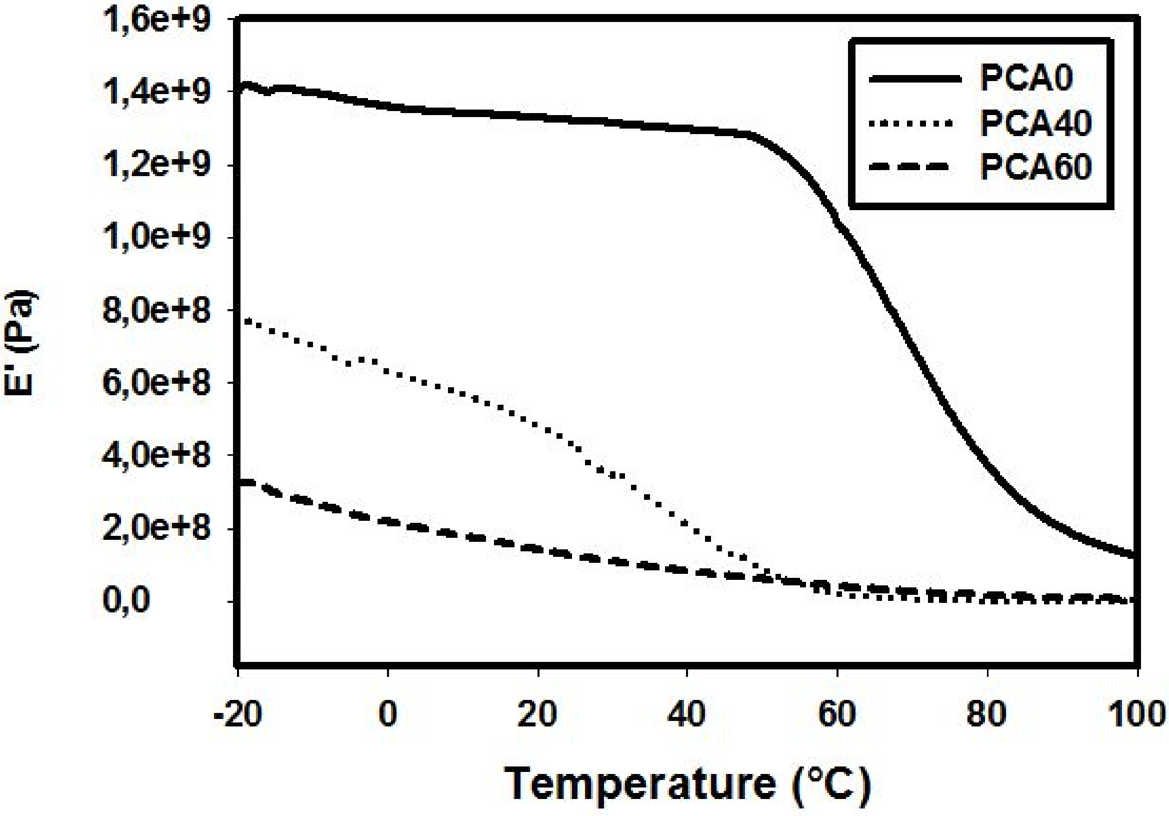

Figure S3. Storage modulus plots of the AzSBO-containing PCA films.
